# Sociodemographic and Lifestyle Factors Associated with Cardiovascular Risk in a Large Cohort of Spanish Workers

**DOI:** 10.5041/RMMJ.10555

**Published:** 2025-10-31

**Authors:** Joan Obrador de Hevia, Ángel Arturo López-González, José Ignacio Ramírez-Manent, Carla Busquets-Cortés, Pedro Juan Tárraga López, Miguel García Samuelsson, Pere Riutord-Sbert

**Affiliations:** 1 ADEMA-Health Group, University Institute of Health Sciences (IUNICS), Palma, Spain; 2 Faculty of Dentistry, ADEMA-UIB University School, Palma, Spain; 3 Balearic Islands Health Service, Palma, Spain; 4 Balearic Islands Health Research Institute Foundation (IDISBA), Palma, Spain; 5 Faculty of Medicine, University of the Balearic Islands, Palma, Spain; 6 Faculty of Medicine, University of Castilla-La Mancha, Albacete, Spain

**Keywords:** Alcohol consumption, cardiovascular risk, Mediterranean diet, occupational health, physical activity, REGICOR, SCORE2, sociodemographic factors

## Abstract

**Background:**

Cardiovascular disease is the leading global cause of death, with lifestyle and sociodemographic factors playing key roles in cardiovascular risk (CVR).

**Objective:**

This two-phase study assessed the associations of alcohol intake, Mediterranean diet adherence, physical activity, and sociodemographic variables with CVR—as measured by the Registre Gironí del Cor (REGICOR) function and Systematic COronary Risk Evaluation 2 (SCORE2) algorithm—in a large cohort of Spanish workers (Phase 1). A secondary aim was to examine CVR trends from 2010 to 2020 (Phase 2).

**Methods:**

A two-phase study was conducted: a cross-sectional analysis of 139,634 workers (Phase 1) and a longitudinal follow-up of 40,431 participants (Phase 2). Anthropometric, clinical, biochemical, and behavioral data were collected using standardized procedures. Multinomial logistic regression was used to evaluate associations.

**Results:**

Phase 1 results showed a higher CVR associated with male sex, older age, lower education, manual labor, smoking, physical inactivity, low adherence to the Mediterranean diet, and alcohol consumption. In Phase 2, CVR increased over the decade, especially among smokers, sedentary individuals, and those with lower education.

**Conclusions:**

Both modifiable behaviors and structural determinants significantly influence CVR. Preventive strategies should integrate lifestyle promotion with measures to reduce social inequalities, with targeted actions for vulnerable groups.

## INTRODUCTION

Cardiovascular disease remains the leading cause of morbidity and mortality worldwide, accounting for an estimated 17.9 million deaths annually, or approximately 32% of all global deaths according to the World Health Organization.^1^ While advances in medical treatment have contributed to improvements in prognosis and life expectancy, the burden of cardiovascular risk (CVR) factors continues to rise, particularly in working-age populations. In this context, identifying modifiable behavioral determinants and their interactions with structural variables such as sex, educational attainment, and social class is essential for designing effective public health strategies.

Cardiovascular diseases remain the leading cause of mortality in Spain, accounting for 26.4% of all deaths in 2022, with ischemic heart disease and cerebrovascular disease being the most prevalent subtypes.^2^ Similar trends are observed across Europe, where cardiovascular disease is responsible for nearly 4 million deaths annually, representing 37% of all deaths in the region.^3^ These figures highlight the urgent need for large-scale epidemiological studies that can identify risk factors and inform preventive strategies.

Epidemiological evidence consistently highlights that lifestyle behaviors—including smoking, alcohol consumption, diet quality, and physical activity—play a critical role in the development of cardiometabolic conditions and ultimately in CVR.^4^,^5^ Among these, alcohol consumption has long been a controversial factor, with some studies suggesting potential cardioprotective effects of moderate intake, while, in contrast, others underscore its deleterious impact on blood pressure, lipid profiles, and arrhythmias, especially when consumption exceeds low-to-moderate thresholds.^6^,^7^ The relationship between alcohol and cardiovascular health is further complicated by its strong correlation with socioeconomic status, cultural norms, and gender-related behaviors.^8^

In addition to alcohol, the Mediterranean diet—rich in fruits, vegetables, whole grains, legumes, fish, and olive oil—has garnered substantial attention for its cardioprotective properties. Numerous cohort studies and randomized clinical trials, including the landmark PREDIMED trial, have demonstrated that adherence to this dietary pattern significantly reduces the incidence of cardiovascular events and improves metabolic parameters.^9^–^11^ Likewise, regular physical activity is well established as a cornerstone of cardiovascular disease prevention, exerting beneficial effects on blood pressure, insulin sensitivity, lipid metabolism, and inflammation.^12^–^14^ The co-existence and interplay of these health behaviors with sociodemographic factors, however, remain insufficiently explored in large occupational cohorts.

The occupational environment is a crucial setting for monitoring and addressing CVR. Workers are exposed to specific risk factors related not only to job strain, shift work, and physical workload, but also to their sociodemographic context and behavioral patterns. Previous studies in occupational health have shown that employees with lower educational levels and social class are more likely to engage in unhealthy lifestyles, accumulate cardiometabolic risk factors, and have poorer access to preventive healthcare.^15^,^16^ Therefore, examining the joint association of sociodemographic characteristics and lifestyle habits with CVR in working populations can offer valuable insights for tailored prevention programs.

Cardiovascular risk estimation tools are widely used to identify individuals at high risk of future events and to guide therapeutic decisions. The Registre Gironí del Cor (REGICOR) function, an adaptation of the Framingham equation calibrated for the Spanish population, estimates the 10-year risk of coronary events by integrating traditional risk factors, including age, sex, smoking, blood pressure, and lipid profile.^17^ In contrast, the newer Systematic COronary Risk Evaluation 2 (SCORE2) algorithm, developed by the European Society of Cardiology, predicts the 10-year risk of cardiovascular morbidity and mortality and incorporates updated European epidemiological data.^18^ Both tools serve as complementary approaches for stratifying CVR in epidemiological studies and clinical practice.

Despite the relevance of these instruments, few studies have examined how modifiable lifestyle behaviors such as alcohol consumption, physical activity, and dietary adherence influence REGICOR and SCORE2 scores in large, real-world cohorts. Moreover, there is a paucity of data exploring sex-specific associations and temporal trends in CVR across subgroups defined by social class and educational attainment. Understanding how these factors interact over time is crucial for addressing persistent health inequalities and evaluating the effectiveness of public health interventions at the population level.

In this context, the present study aims to analyze the association of alcohol consumption, other healthy habits (Mediterranean diet and physical activity), and sociodemographic variables with the values of different CVR scales (REGICOR and SCORE2) in a large sample of 139,634 Spanish workers. The objectives of Phase 1 were to assess the association of CVR factors as calculated by REGICOR and SCORE2 equations with sex, sociodemographic characteristics, and lifestyle behaviors. The objective of Phase 2 was to assess the temporal evolution (2010–2020) of CVR in relation to key lifestyle and structural determinants, stratified by sex.

By leveraging a large occupational cohort with standardized clinical and lifestyle data, this study provides an opportunity to comprehensively assess the real-world impact of modifiable and non-modifiable determinants on cardiovascular health. The findings are expected to contribute to a better understanding of how health behaviors and structural inequalities shape CVR profiles in the working population, and to inform targeted preventive strategies aimed at reducing cardiovascular disease burden in both males and females.

In addition, this study explores the complex interplay between social class and education, encompassing both material and cognitive dimensions of socioeconomic position, as well as cardiovascular health outcomes. This dual approach recognizes that lower socioeconomic status may not only constrain access to healthy lifestyle options but may also influence health literacy, health beliefs, and self-efficacy regarding behavior change.^19^,^20^ These social gradients are further accentuated by gender norms that differentially affect the adoption of health behaviors such as alcohol use, diet, and exercise.^21^

Notably, the inclusion of temporal trends in CVR estimation (comparing data from 2010 and 2020) allows for the evaluation of how lifestyle changes and public health initiatives may have influenced the evolution of cardiovascular profiles over time. The observed trends will be crucial to assess the effectiveness—or unintended gaps—of health promotion campaigns, especially in the context of increasing social inequalities and changing consumption patterns.

In sum, the current study addresses a relevant gap in the cardiovascular epidemiology literature by jointly analyzing alcohol intake, diet, physical activity, and structural factors in relation to CVR estimation in a large occupational cohort. The evidence generated has the potential to inform workplace health policies, guide targeted interventions, and ultimately contribute to the reduction of CD burden in Spain and similar settings.

## METHODS

A two-phase study design was employed. The first phase involved a descriptive, cross-sectional analysis of 139,634 employees from multiple regions across Spain, encompassing a broad spectrum of occupational sectors (56,352 females and 83,282 males). Participants were recruited during routine occupational health evaluations conducted by the collaborating companies’ healthcare services.

The second phase consisted of a retrospective longitudinal analysis conducted on a subsample of 40,431 individuals (24,229 males and 16,202 females) drawn from the original cohort, covering a ten-year observation period from 2010 to 2020.

All clinical, anthropometric, and biochemical data were collected by qualified healthcare professionals associated with the participating organizations. To ensure consistency and reduce inter-observer variability, standardized measurement protocols were established and implemented prior to data acquisition.

Eligibility criteria for inclusion in the study were as follows:

- Age between 18 and 69 years, representing the economically active population;
- Current employment status within one of the enrolled companies and absence of temporary work disability at the time of data collection;
- Complete availability of all variables required to compute CVR scores;
- Voluntary agreement to participate and authorization for the use of anonymized data in epidemiological analyses;
- For inclusion in the longitudinal analysis, the availability of complete records from both 2009 and 2019, with no major changes reported in sociodemographic attributes or lifestyle behaviors across the study period.

**Figure 1:**
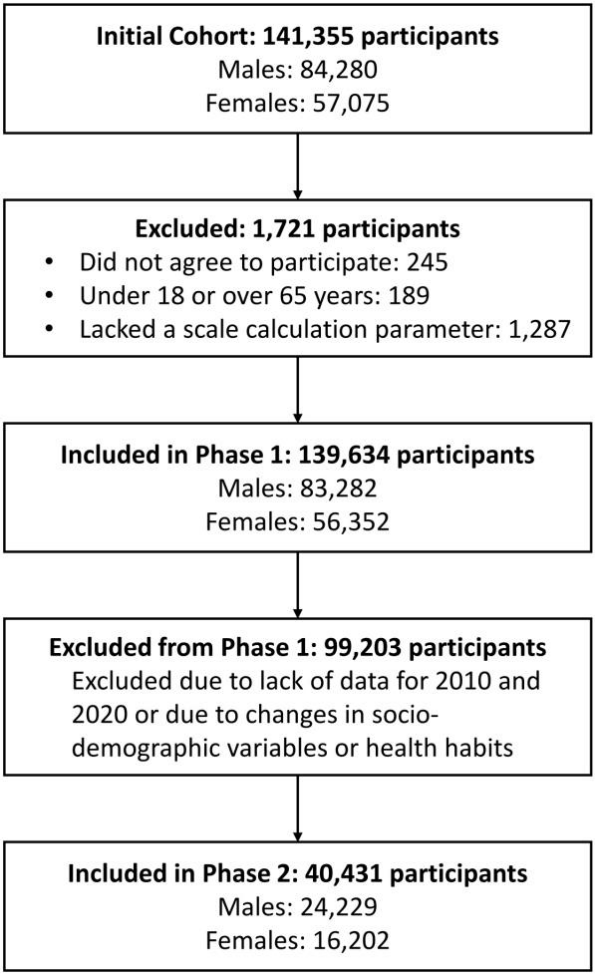
Flow Chart Reflecting Study Participant Selection.

An overview of the participant selection process is provided in Figure 1, which clearly visualizes the participant selection process, ensuring methodological transparency. The exclusion of individuals is adequately justified, enhancing the study’s internal validity.

### Variable Assessment and Data Collection Procedures

Following prior standardization of measurement protocols, clinical, anthropometric, and laboratory assessments were conducted by trained medical and nursing personnel affiliated with the study. These assessments included waist circumference, body weight, height, blood pressure, and biochemical parameters.

Body weight and height were measured using a SECA 700 calibrated scale (seca GmbH & Co. KG, Hamburg, Germany). Waist circumference was measured using a SECA measuring tape (seca GmbH & Co. KG, Hamburg, Germany), with participants standing upright, feet together, arms relaxed, and abdomen uncontracted. The tape was placed horizontally, encircling the midpoint between the iliac crest and the lower margin of the last palpable rib, in accordance with established anthropometric guidelines.^22^

Blood pressure was measured using a calibrated automatic sphygmomanometer (OMRON M3; OMRON Healthcare Co., Ltd, Kyoto, Japan). Measurements were taken with the participant seated and after a rest period of at least 10 minutes. Three consecutive readings were obtained at 1-minute intervals, and the average value was used for analysis.^23^

Fasting blood samples were collected after a minimum of 12 hours without food intake. Biochemical analyses included total cholesterol, plasma glucose, and triglyceride levels, all of which were determined using a standardized enzymatic autoanalyzer. High-density lipoprotein cholesterol (HDL-C) levels were determined via precipitation with dextran sulfate–magnesium chloride. Low-density lipoprotein cholesterol (LDL-C) was estimated using the Friedewald formula, as follows:


LDL=Total cholesterol-HDL-(Triglycerides/5).

All lipid and glucose values were reported in mg/dL.^24^

Cardiovascular risk was assessed using two predictive models specifically adapted to the Spanish population: REGICOR and SCORE2.

The REGICOR equation represents a recalibrated version of the original Framingham risk score. It was specifically adapted to the epidemiological characteristics of the Spanish population, with calibration based on cohort data from Catalonia. This adjustment was necessary because the original Framingham model tended to overestimate CVR in countries such as Spain, where the prevalence of cardiovascular disease is significantly lower than that observed in the United States.^25^ The REGICOR scale estimates the 10-year risk of experiencing a coronary event by considering classical risk factors such as age, sex, smoking status, systolic blood pressure, total cholesterol, HDL cholesterol, and diabetes mellitus. The score expresses 10-year coronary risk as a percentage or in categories: low (<5%), moderate (5%–9.9%), and high (≥10%).

Despite the availability of REGICOR, current clinical practice in Spain has increasingly favored the use of the SCORE (Systematic COronary Risk Estimation) system, particularly the updated SCORE2 algorithm.^26^ The SCORE2 system estimates the 10-year probability of both fatal and non-fatal atherosclerotic cardiovascular events in individuals aged 40 to 69 years. This model is calibrated using national-level cardiovascular mortality data published by the World Health Organization, and it is stratified into four distinct risk categories, corresponding to different levels of CVR across European regions. Spain is classified within the low-risk category, reflecting the country’s comparatively favorable cardiovascular health profile. The use of SCORE2, therefore, enabled more precise and contextually appropriate risk stratification in Spanish clinical and research settings, enhancing its relevance for preventive cardiology and population health management. It takes into account age, sex, systolic blood pressure, total cholesterol, and smoking status, and applies to individuals without prior cardiovascular disease. The resulting score is expressed as a percentage and categorized as low (<2.5%), moderate (2.5%–4.9%), high (5%–9.9%), and very high (≥10%) cardiovascular risk. These thresholds correspond to countries with intermediate cardiovascular risk, such as Spain.

Participants were classified as current smokers if they reported smoking at least one cigarette per day (or an equivalent form of tobacco use) in the past 30 days or had quit smoking within the previous 12 months.^27^

Dietary habits were assessed using the validated 14-item “Mediterranean Diet Adherence Screener” (MEDAS) from the PREDIMED trial. Each item is scored dichotomously (0 or 1), with a total score of 9 or above indicating high adherence to the Mediterranean dietary pattern, known for its cardioprotective benefits.^28^

Physical activity levels were measured using the International Physical Activity Questionnaire (IPAQ), which captures information about the intensity, duration, and frequency of physical activity over the previous seven days. The questionnaire covers three domains: occupational, transport-related, leisure-time physical activity, and sedentary behavior. Activities are categorized as vigorous, moderate, or walking, and results are expressed in metabolic equivalent task minutes per week (MET-min/week), a standardized metric for comparing activity levels across individuals and populations. The IPAQ is widely recognized for its use in epidemiological and public health research.^29^

Alcohol intake was quantified in standard alcohol units (SAUs), with one SAU equivalent to 10 g of pure ethanol, in accordance with national Spanish guidelines. High alcohol consumption was defined as ≥14 SAUs per week for females and ≥21 SAUs per week for males.^30^

Occupational social class was classified based on the 2011 National Classification of Occupations (CNO-11) and the criteria established by the Spanish Society of Epidemiology. Participants were categorized into three groups:

- Class I: University-educated professionals and senior management positions
- Class II: Skilled self-employed individuals and intermediate-level occupations
- Class III: Manual or unskilled laborers

Educational attainment was categorized into three levels: primary education, secondary education, and university-level education.

### Statistical Analysis

Descriptive statistics were used to summarize the data. For continuous variables, mean values and standard deviations were calculated, and comparisons between groups were performed using the Student *t*-test. The chi-square test was used to assess associations between categorical variables. Multinomial logistic regression modeling was used to determine the relationship between CVR scales and sociodemographic or behavioral factors. All statistical analyses were performed using IBM SPSS Statistics version 29.0 (IBM Corp., Armonk, NY, USA). A two-sided *P*-value less than 0.05 was considered indicative of statistical significance.

## RESULTS

Table 1 outlines the sociodemographic, clinical, and lifestyle characteristics of the study population (*n*= 139,634), stratified by sex. Males exhibited significantly higher values in anthropometric and clinical parameters such as weight, blood pressure, glucose, and triglycerides, whereas females had higher HDL levels and demonstrated higher adherence to healthy behaviors (Mediterranean diet, physical activity, and alcohol abstinence). Educational level, social class, and healthy lifestyle behaviors are well-recognized determinants of individual health. Notably, our analysis revealed significant differences between women and men, with women exhibiting higher values (*P*<0.001), underscoring the importance of sex-specific considerations in health outcomes.

**Table 1:** Study Population Characteristics.

Demographics and Blood Tests	Male (*n*=83,282) Mean±SD	Female (*n*=56,352) Mean±SD	*P*-value
Age (years)	41.4±10.7	40.1±10.4	<0.001

Height (cm)	173.8±7.1	161.2±6.5	<0.001

Weight (kg)	83.2±14.6	66.3±13.9	<0.001

Systolic blood pressure (mmHg)	126.2±15.9	115.6±15.7	<0.001

Diastolic blood pressure (mmHg)	76.6±10.9	71.1±10.7	<0.001

Total cholesterol (mg/dL)	199.6±38.6	194.6±36.9	<0.001

HDL-cholesterol (mg/dL)	50.0±7.7	54.7±9.2	<0.001

LDL-cholesterol (mg/dL)	122.6±37.4	121.5±37.1	<0.001

Triglycerides (mg/dL)	133.8±95.6	90.8±49.7	<0.001

Glucose (mg/dL)	93.0±25.4	86.8±18.1	<0.001

**Specific variables**	**%**	**%**	***P-*** **value**

**Age**			<0.001
<30 years	15.1	18.0	
30–39 years	29.6	31.0	
40–49 years	30.2	30.3	
50–59 years	20.9	17.7	
60–69 years	4.2	3.0	

**Social class**			<0.001
Social class I	7.5	13.6	
Social class II	23.8	32.1	
Social class III	68.7	54.1	

**Education**			<0.001
Elementary school	66.4	48.1	
High school	26.9	40.0	
University	6.7	11.9	

**Smoker**			<0.001
No	66.8	67.9	
Yes	33.2	32.1	

**Physical activity**			<0.001
No	62.4	51.4	
Yes	37.6	48.6	

**Mediterranean diet**			<0.001
No	65.8	52.8	
Yes	34.2	47.2	

**Alcohol consumption**			<0.001
No	67.3	84.4	
Yes	32.7	15.6	

HDL, high-density lipoprotein; LDL, low-density lipoprotein; SD, standard deviation; REGICOR, Registre Gironí del Cor; SCORE2, Systematic COronary Risk Evaluation; SD, standard deviation.

To facilitate the analysis, all variables were transformed into dichotomous categories. Adherence to the Mediterranean diet was defined as a MEDAS questionnaire score of ≥9 points, whereas scores below this threshold were considered indicative of low adherence. Adherence to physical activity recommendations was defined according to compliance with the World Health Organization (WHO) guidelines, corresponding to a classification of moderate or vigorous physical activity based on the IPAQ. Regarding alcohol consumption, participants were classified as drinkers if their intake met the criteria for high alcohol consumption, defined in Spain as ≥14 standard drinking units per week for women and ≥21 units per week for men.

Table 2 presents the mean values of REGICOR and SCORE2 CVR scores across sociodemographic and lifestyle subgroups. Risk scores for both REGICOR and SCORE2 increased with age, lower social class, lower educational level, smoking, physical inactivity, non-adherence to the Mediterranean diet, and alcohol consumption. Similar trends were observed in both males and females. Statistical analyses (Student’s *t*-test, chi-square test, and multinomial logistic regression) confirmed that these associations were significant (*P*<0.05). These results confirm that cardiovascular risk increases with age, lower educational attainment, disadvantaged social class, smoking, physical inactivity, low adherence to the Mediterranean diet, and alcohol consumption.

**Table 2:** Mean Values of Different Cardiovascular Risk Scales According to Sociodemographic Variables and Healthy Habits by Sex.

Variables	All		Males		Females	

	REGICOR	SCORE2	REGICOR	SCORE2	REGICOR	SCORE2
	Mean±SD	Mean±SD	Mean±SD	Mean±SD	Mean±SD	Mean±SD
**Age**						
30–39 years	1.8±1.0	No data	2.2±1.0	No data	1.1±0.5	No data
40–49 years	2.6±1.6	0.5±0.7	3.0±1.7	0.7±0.8	2.0±1.3	0.1±0.2
50–59 years	4.5±2.6	1.8±1.7	4.9±2.8	2.5±1.7	3.8±2.0	0.6±0.7
60–69 years	6.1±3.0	4.1±3.1	6.6±3.1	5.1±3.1	5.2±2.3	1.8±1.2

**Social class**						
Social class I	2.5±2.1	0.9±1.4	3.3±2.3	1.4±1.7	1.7±1.4	0.2±0.4
Social class II	2.8±2.2	1.0±1.6	3.4±2.4	1.6±1.8	2.1±1.7	0.3±0.6
Social class III	3.3±2.4	1.3±1.8	3.7±2.5	1.8±2.0	2.7±2.0	0.4±0.7

**Education**						
Elementary school	3.4±2.4	1.3±1.7	3.8±2.7	1.7±1.9	2.7±1.9	0.4±0.7
High school	2.9±2.2	1.1±1.7	3.5±2.3	1.7±2.0	2.2±1.8	0.3±0.6
University	2.5±1.9	0.9±1.5	3.3±2.4	1.4±1.7	1.6±1.3	0.2±0.4

**Smoker**						
No	2.8±2.0	0.9±1.3	3.1±2.0	1.3±1.5	2.3±1.8	0.3±0.6
Yes	3.8±2.8	1.8±2.2	4.6±2.9	2.6±2.5	2.6±2.0	0.5±0.8

**Physical activity**						
No	3.6±2.5	1.4±1.8	4.0±2.6	1.9±2.0	2.9±2.1	0.5±0.8
Yes	2.2±1.5	0.7±1.3	2.6±1.6	1.2±1.6	1.6±1.2	0.2±0.5

**Mediterranean diet**						
No	3.6±2.5	1.3±1.8	3.9±2.6	1.8±2.0	2.8±2.1	0.4±0.7
Yes	2.1±1.5	0.7±1.3	2.6±1.6	1.3±1.6	1.7±1.3	0.2±0.5

**Alcohol consumption**						
No	2.6±1.9	0.9±1.5	3.1±2.0	1.5±1.8	2.0±1.4	0.3±0.6
Yes	4.2±2.8	1.6±1.9	4.3±2.8	2.0±2.1	3.9±2.5	0.6±0.9

* Complete data were not available for all subjects; denominators provided in the supplementary table. All differences were statistically significant (*P*<0.05).

REGICOR, Registre Gironí del Cor; SCORE2, Systematic COronary Risk Evaluation; SD, standard deviation.

Table 3 reports the prevalence of moderate-high REGICOR and medium-high SCORE2 risk levels. A higher burden was found among males, individuals with lower educational attainment and social class, and those engaging in unhealthy behaviors such as smoking, sedentarism, or poor diet. The protective role of physical activity, Mediterranean diet, and alcohol abstinence was evident in both sexes. Using multiple risk scales enabled a more nuanced assessment, emphasizing the value of multidimensional approaches in population studies.

**Table 3:** Prevalence^*^ of Increased Rates of Different Cardiovascular Risk Scales According to Sociodemographic Variables and Healthy Habits by Sex.

Variables	Males		Females	

	REGICOR Moderate-High %	SCORE2 Medium-High %	REGICOR Moderate-High %	SCORE2 Medium-High %
**Age**				
<30 years	No data	No data	No data	No data
30–39 years	2.5	No data	0.0	No data
40–49 years	14.9	0.7	3.5	0.0
50–59 years	44.8	16.7	29.0	0.3
60–69 years	73.4	58.6	63.1	6.3

**Social class**				
Social class I	17.6	8.6	5.1	0.1
Social class II	21.7	9.7	7.9	0.3
Social class III	25.8	12.1	15.7	0.7

**Education**				
Elementary school	26.9	11.6	15.2	0.7
High school	23.5	11.2	9.7	0.3
University	18.3	8.9	4.4	0.1

**Smoker**				
No	17.3	7.3	11.6	0.3
Yes	39.5	20.2	12.3	0.9

**Physical activity**				
No	30.0	12.7	18.0	0.7
Yes	9.9	6.8	3.2	0.2

**Mediterranean diet**				
No	29.0	12.4	17.4	0.6
Yes	10.2	7.2	3.7	0.2

**Alcohol consumption**				
No	17.3	9.0	6.4	0.3
Yes	34.5	13.9	33.7	1.0

* Complete data were not available for all subjects; denominators provided in the supplementary table. All differences were statistically significant (*P*<0.05).

REGICOR, Registre Gironí del Cor; SCORE2, Systematic COronary Risk Evaluation; SD, standard deviation.

Table 4 provides adjusted odds ratios for moderate-high CVR (REGICOR) and medium-high risk (SCORE2). The model showed strong associations between elevated risk and factors such as older age, lower educational attainment, disadvantaged social class, smoking, physical inactivity, poor diet adherence, and alcohol consumption. Males exhibited significantly higher odds across both scales. These findings underscore the need for targeted preventive interventions focused on vulnerable groups and modifiable lifestyle factors.

**Table 4:** Multinomial Logistic Regression.

Variables	REGICOR, Moderate–High OR (95% CI)	SCORE2, Medium–High OR (95% CI)
**Sex**		
Females	1	1
Males	2.31 (2.21–2.42)	4.21 (3.98–4.44)

**Age**		
30–39 years	1	None
40–49 years	4.86 (4.53–5.22)	1
50–59 years	8.91 (8.11–9.72)	5.66 (5.01–6.32)
60–69 years	31.35 (29.02–33.87)	56.10 (53.90–58.31)

**Social class**		
Social class I	1	1
Social class II	1.51 (1.41–1.52)	1.23 (1.19–1.27)
Social class III	1.58 (1.49–1.68)	1.48 (1.39–1.58)

**Education**		
Elementary school	1	1
High school	1.23 (1.20–1.27)	1.30 (1.24–1.36)
University	1.50 (1.41–1.60)	1.51 (1.43–1.60)

**Smoker**		
No	1	1
Yes	4.87 (4.17–5.58)	3.18 (2.95–3.42)

**Physical activity**		
No	1	1
Yes	2.58 (2.32–2.83)	2.14 (1.97–2.32)

**Mediterranean diet**		
No	1	1
Yes	1.91 (1.68–2.15)	1.88 (1.60–2.16)

**Alcohol consumption**		
No	1	1
Yes	2.20 (2.10–2.30)	2.45 (2.29–2.62)

CI, confidence interval; OR, odds ratio; REGICOR, Registre Gironí del Cor; SCORE2, Systematic COronary Risk Evaluation 2.

Table 5 examines the percentage change in CVR prevalence between 2010 and 2020. A general increase is noted, particularly among male smokers, sedentary individuals, and those with lower educational levels. The most pronounced increases occurred in individuals with multiple combined risk factors. These findings raise concerns about the worsening trends in cardiovascular health over the past decade, suggesting a stagnation or reversal in primary prevention efforts.

**Table 5:** Temporal Evolution of Moderate-High REGICOR and Medium-High SCORE2 Cardiovascular Risk Between 2010 (PRE) and 2020 (POST), by Sociodemographic and Lifestyle Variables and Sex.

Variables	Males (*n*=24,229)			Females (*n*=16,202)		

	*N*	REGICOR Moderate-High % Pre-Post (Change)	SCORE2 Medium-High % Pre-Post (Change)	*N*	REGICOR Moderate-High % Pre-Post (Change)	SCORE2 Medium-High % Pre-Post (Change)
**Social Class**						
Social class I	1,900	18.0–19.9 (+10.6)	8.5–9.2 (+8.2)	2,128	4.9–5.3 (+8.2)	4.8–5.3 (+8.8)
Social class II	5,769	22.5–25.9 (+15.3)	9.1–10.6 (+16.5)	5,290	7.5–8.3 (+11.3)	7.8–8.8 (+12.4)
Social class III	16,560	25.9–31.1 (+19.9)	12.2–14.9 (+22.1)	8,784	15.6–17.8 (+14.1)	11.9–14.0 (+17.9)

**Education**						
Elementary school	16,022	23.4–27.8 (+18.9)	11.7–14.3 (+22.2)	7,836	15.3–17.5 (+14.3)	12.3–14.5 (+17.6)
High school	6,501	22.9–26.6 (+18.2)	9.3–10.9 (+17.2)	6,518	9.2–10.2 (+11.2)	7.5–8.5 (+12.8)
University	1,706	18.9–20.9 (+10.4)	8.8–10.6 (+20.5)	1,848	3.8–4.1 (+8.0)	4.6–5.0 (+9.2)

**Smoker**						
No	16,244	17.6–18.9 (+8.3)	7.0–7.7 (+10.0)	10,992	4.4–4.7 (+7.9)	5.8–6.4 (+9.4)
Yes	7,985	40.0–50.1 (+25.2)	20.7–28.6 (+38.2)	5,210	21.7–25.8 (+18.8)	21.3–25.8 (+21.3)

**Physical activity**						
No	15,045	30.3–37.4 (+23.5)	12.6–16.6 (+31.7)	8,327	17.9–19.3 (+17.5)	20.8–25.4 (+21.9)
Yes	9,184	10.1–11.0 (+8.5)	6.6–7.5 (+13.6)	7,875	3.2–3.5 (+7.7)	4.1–4.3 (+5.4)

**Mediterranean diet**						
No	15,866	27.1–33.3 (+22.8)	12.3–16.1 (+30.9)	8,632	17.4–20.3 (+16.8)	19.7–23.8 (+20.8)
Yes	8,363	11.1–12.3 (+10.5)	6.9–7.9 (+14.5)	7,570	3.5–3.8 (+8.4)	5.6–6.0 (+7.2)

**Alcohol consumption**						
No	16,258	10.4–11.6 (+11.9)	8.7–10.0 (+14.9)	13,707	6.1–6.6 (+7.8)	8.5–9.4 (+10.4)
Yes	7,971	34.8–42.4 (+21.8)	14.0–20.6 (+47.1)	2,495	34.5–43.4 (+25.9)	18.6–22.3 (+19.9)

PRE year, 2010; POST year, 2020; REGICOR, Registre Gironí del Cor; SCORE2, Systematic COronary Risk Evaluation.

## DISCUSSION

This study examined the associations of alcohol consumption, adherence to the Mediterranean diet, physical activity, and sociodemographic factors with CVR, estimated using the REGICOR and SCORE2 equations in a large cohort of Spanish workers. A retrospective analysis (Phase 2) was also performed to assess temporal trends over a decade. The findings highlight the cumulative and independent effects of behavioral and structural factors on CVR, underlining the need for multidimensional prevention strategies.

The role of alcohol in cardiovascular health has long been debated. Epidemiological data often suggest a J-shaped curve, with low-to-moderate intake associated with reduced risk and higher consumption linked to adverse outcomes.^31^ In the present study, individuals with moderate alcohol intake exhibited slightly higher CVR compared to abstainers, but this association was modified by other lifestyle factors. Similar findings were observed in a recent large-scale meta-analysis that emphasized the complex interaction between alcohol, diet, and metabolic status.^32^

Adherence to a Mediterranean dietary pattern was strongly associated with reduced CVR in both males and females. This result is consistent with multiple prior investigations, including randomized controlled trials, which have demonstrated significant cardiovascular benefits of the Mediterranean diet— particularly when enriched with olive oil or nuts.^33^ Mechanistically, the diet exerts anti-inflammatory, antioxidant, and lipid-modulating effects,^34^,^35^ and our results reinforce its utility as a non-pharmacological intervention for CVR reduction in the workplace.

Our findings confirm that regular physical activity is a key protective factor against CVR, with a dose-response association observed across both risk equations. Participants who reported higher activity levels had substantially lower REGICOR and SCORE2 scores. These findings are consistent with global evidence showing that physical inactivity is responsible for more than 5 million deaths annually due to non-communicable diseases.^36^,^37^ Promoting exercise, even in the form of active commuting or workplace interventions, remains a public health priority.

The analysis revealed robust associations between low educational attainment, disadvantaged occupational class, and increased CVR. Individuals in social class III and those with only primary education exhibited higher REGICOR and SCORE2 scores compared to their more privileged counter-parts. These findings support a growing body of literature indicating that socioeconomic status is a major determinant of health, independent of traditional risk factors.^38^,^39^ Lower socioeconomic status is often linked to reduced access to healthcare, limited health literacy, and higher exposure to behavioral risks.^40^

Males consistently showed higher CVR scores across both risk scales, aligning with international data indicating that males generally develop cardiovascular conditions earlier than females. However, under-recognition of cardiovascular disease in females, particularly in occupational settings, remains a challenge. The SCORE2 algorithm’s sex-specific calibration enabled more accurate risk stratification; however, interventions should also account for sex-related patterns in health-seeking behavior and risk perception.^41^,^42^

The longitudinal component of this study showed concerning increases in CVR over the 10-year observation period, particularly among smokers, individuals with poor diet adherence, and sedentary workers. These trends may reflect broader societal shifts in health behaviors, increased psychosocial stress in working populations, and the partial efficacy of population-level interventions.^43^ These patterns mirror those reported in recent European surveillance data, which indicate a stagnation in cardiovascular disease mortality reductions in several low-risk countries.^44^,^45^

The use of multinomial logistic regression revealed that the combination of multiple unhealthy behaviors compounded CVR estimates. Smoking was the strongest predictor, followed by physical inactivity and poor adherence to the Mediterranean diet. Alcohol consumption remained a significant but less potent independent predictor. This cumulative effect aligns with the findings of the INTERHEART study, which reported that nine modifiable risk factors account for over 90% of the population-attributable risk for myocardial infarction worldwide.^46^

Our findings reinforce the need for integrated prevention strategies that combine promotion of healthy lifestyle behaviors with policies addressing social determinants of health. Workplace interventions, community-based programs, and policies that improve access to healthy food and safe environments for physical activity are crucial to reduce the burden of cardiovascular disease.^47^,^48^

### Contribution of this Study

Although our results are consistent with previous evidence, the novelty of this study lies in the large-scale analysis of over 139,000 Spanish workers, with detailed stratification by sex, age, and socioeconomic status. This approach provides unique insights into occupational and social determinants of CVR within a Mediterranean population.

The broader implications of our findings extend beyond Spain. Cardiovascular disease remains the leading cause of mortality worldwide, and the risk patterns observed in this Mediterranean working population are relevant to other European and high-income countries with similar occupational and social structures. Our results underscore the need for workplace health promotion programs and public health policies that address both individual lifestyle behaviors and structural determinants of cardiovascular health at an international level.^2^–^4^

Widespread promotion of the Mediterranean diet throughout Western countries could substantially reduce cardiovascular risk. To achieve meaningful impact, this strategy should be pursued at all levels, including workplace and school programs, public media campaigns, and coordinated political and healthcare initiatives.

### Strengths and Limitations

This study possesses several notable strengths that contribute to its scientific rigor and relevance. First and foremost, the sample size is exceptionally large, encompassing 139,634 working individuals from diverse regions of Spain and representing a wide range of occupational sectors. This robust cohort enhances the generalizability of the findings to the broader working population and offers adequate statistical power to detect even small differences across subgroups.

Second, the study integrates both cross-sectional and longitudinal components, with a 10-year follow-up for a substantial subsample (*n*=40,431). This dual-phase design allows not only for the identification of associations between lifestyle and structural factors with CVR, but also for the examination of temporal trends in risk evolution over a decade. The inclusion of repeated measures in a stable occupational cohort strengthens causal inferences and provides valuable insight into the effectiveness—or lack thereof—of public health policies over time.

Third, CVR was estimated using two validated and widely used tools—REGICOR and SCORE2—both adapted to the Spanish and European contexts, respectively. The concurrent application of these scales allows for a more nuanced stratification of CVR and supports the robustness of the results, as consistency across tools provides greater confidence in the observed associations.

Fourth, the use of standardized protocols for clinical, anthropometric, and laboratory measurements by trained healthcare professionals across participating companies ensures high-quality data and minimizes inter-observer variability. Furthermore, the incorporation of validated questionnaires for dietary assessment (PREDIMED), physical activity (IPAQ), and alcohol consumption strengthens the reliability of lifestyle-related variables.

Finally, the study’s analytic approach, including the use of multinomial logistic regression models adjusted for relevant covariates, supports a comprehensive assessment of the independent contributions of sociodemographic and behavioral factors to CVR. The stratification by sex throughout the analysis is also a key methodological strength, as it accounts for known differences in cardiovascular physiology and risk profiles between males and females.

Despite these strengths, several limitations must be acknowledged. The observational nature of the study precludes definitive causal inferences. While associations were robust and consistent with the existing literature, residual confounding by unmeasured variables—such as psychosocial stress, sleep quality, or family history—may still influence the results.

The study population consists exclusively of currently employed individuals, which may introduce a “healthy worker effect.” Those with severe health conditions or disabilities may be underrepresented, potentially leading to an underestimation of true CVR in the general population. This selection bias may limit the external validity of the findings to unemployed, disabled, sick, or retired individuals.^49^

Medication data (such as use of statins, antihypertensives, or antidiabetic drugs) were not collected in our cohort. This limitation may result in an underestimation of CVR factors, as treated individuals could present normal or near-normal values despite underlying pathology. This omission could attenuate or exaggerate observed associations, depending on treatment distribution across sociodemographic groups.^50^,^51^

While the longitudinal subsample allowed for an assessment of risk evolution over time, changes in behavior, medication use, or health awareness during the follow-up period may not have been fully captured, particularly if such changes occurred between assessment points or were not sustained.

Several variables, including diet, physical activity, and alcohol intake, were based on self-reported questionnaires. This approach may introduce recall bias and social desirability bias, potentially leading to under- or overestimation of certain lifestyle behaviors. Although validated instruments were used, misclassification of exposure remains possible, particularly in sensitive domains such as alcohol intake. These limitations are common in large-scale epidemiological studies but should be considered when interpreting our results.^52^,^53^

In the longitudinal analyses, some degree of loss to follow-up occurred, which may have introduced attrition bias and reduced representativeness. Additionally, socioeconomic information was limited to occupational social class, and more detailed indicators such as income, wealth, or neighborhood deprivation were not available. This limitation restricts a more nuanced assessment of the impact of social determinants on CVR.^54^,^55^

Given the observational design of our study, causal relationships between exposures and cardiovascular outcomes cannot be established. Our results should therefore be interpreted as associations, in line with the inherent limitations of cross-sectional and cohort analyses.^56^

Finally, the REGICOR and SCORE2 equations, while validated, are still probabilistic estimators based on population data and may not fully capture individual risk. Moreover, SCORE2’s performance in younger individuals and in ethnically diverse populations may require further evaluation, even within low-risk countries such as Spain.

In summary, the present study is distinguished by its large and diverse sample, dual risk estimation models, comprehensive lifestyle and sociodemographic data, and longitudinal follow-up. However, caution is warranted in interpreting the findings due to inherent limitations related to observational design, self-reported data, and potential selection bias. Future research incorporating clinical outcomes, medication data, and qualitative insights into behavioral change would provide a more complete understanding of CVR trajectories in working populations.

## CONCLUSIONS

In conclusion, this study underscores the significant influence of modifiable lifestyle factors—namely, alcohol intake, dietary habits, and physical activity—as well as structural determinants such as sex, educational level, and occupational social class on CVR in a large sample of Spanish workers. The observed increase in CVR over time among high-risk subgroups suggests the need for more intensive, equity-oriented preventive strategies in the workplace and broader community. Interventions tailored to sociodemographic profiles and health behaviors may be the most effective approach to reducing cardiovascular disease burden and health inequalities.

## Supplementary Information



## Abbreviations

CVR: cardiovascular risk
IPAQ: International Physical Activity Questionnaire
REGICOR: Registre Gironí del Cor
SAU(s): standard alcohol unit(s)
SCORE2: Systematic COronary Risk Evaluation 2.
