# Supplementary material for: Sociodemographic and Lifestyle Factors Associated with Cardiovascular Risk in a Large Cohort of Spanish Workers
Source: Rambam Maimonides Med J. 2025 Oct 31;16(4):e0020. doi: 10.5041/RMMJ.10555 (PMC12591517; doi:10.5041/RMMJ.10555)
Supplement: Supplementary file 1 [file rmmj-16-4-e0020_Supplement.pdf]

SUPPLEMENTARY MATERIAL

This appendix has been provided by the authors for the benefit of readers

Supplement to Sociodemographic and Lifestyle Factors Associated with Cardiovascular Risk in a Large Cohort of Spanish Workers

Obrador de Hevia J, López-González ÁA, Ramírez-Manent JI, Busquets-Cortés C, López JT, Samuelsson MG, Riutord-Sbert P. Sociodemographic and Lifestyle Factors Associated with Cardiovascular Risk in a Large Cohort of Spanish Workers. Rambam Maimonides Med J 2025;16 (4):e0020. doi:10.5041/RMMJ.10555

EXPLANATION OF THE DATA USED TO CALCULATE TABLES 2 AND 3

Missing data in supplementary Table S1 (next page) are mainly due to the application criteria of the cardiovascular risk equations and incomplete information in certain self-reported variables. Specifically, the SCORE2 algorithm was only applicable to participants aged 40–69 years, which explains the absence of data for younger age groups. In addition, some participants had incomplete responses for lifestyle variables such as physical activity, dietary habits, and alcohol consumption, which were collected through self-administered questionnaires. No data imputation was performed; therefore, analyses were based on available cases only. The overall proportion of missing data was low and randomly distributed across sociodemographic categories, minimizing the likelihood of systematic bias.

Table S1. Data Used for Calculating Tables 2 and 3.

Variables	Number of Subjects Evaluated			
	Males (*n*=83,282)		Females (*n*=56,352)	
	REGICOR	SCORE2	REGICOR	SCORE2
**Age**				
30-39 years	13,220	No data	8,980	No data
40-49 years	25,178	25,178	17,094	17,094
50-59 years	17,370	17,370	9,984	9,984
60-69 years	3,528	3,528	1,704	1,704
**Social class**				
Social class I	4,210	3,034	3,926	2,548
Social class II	15,470	12,266	12,014	8,544
Social class III	39,616	30,568	21,822	17,576
**Education**				
Elementary school	38,134	29,252	19,742	15,964
High school	17,282	13,760	14,558	10,476
University	3,880	2,856	3,462	2,228
**Smoker**				
No	40,980	31,942	26,192	19,914
Yes	18,316	13,926	11,570	8,754
**Physical activity**				
No	42,156	34,298	22,620	18,656
Yes	17,140	11,570	15,142	10,012
**Mediterranean diet**				
No	44,070	35,640	23,100	18,942
Yes	15,226	10,228	14,662	9,726
**Alcohol consumption**				
No	35,680	25,552	29,822	21,532
Yes	23,616	20,316	7,940	7,136

REGICOR, Registre Gironí del Cor; SCORE2, Systematic COronary Risk Evaluation 2; SD, standard deviation.
